# HinT proteins and their putative interaction partners in *Mollicutes *and *Chlamydiaceae*

**DOI:** 10.1186/1471-2180-5-27

**Published:** 2005-05-18

**Authors:** Miriam Hopfe, Johannes H Hegemann, Birgit Henrich

**Affiliations:** 1 Institute of Medical Microbiology, Moorenstrasse 5, 40225 Duesseldorf, Germany; 2 Chair of Functional Genome Research of Microorganisms, University Street 1, Heinrich-Heine-University, 40225 Duesseldorf, Germany; 3 Center of Biological and Clinic Research, University Street 1, Heinrich-Heine-University, 40225 Duesseldorf, Germany

## Abstract

**Background:**

HinT proteins are found in prokaryotes and eukaryotes and belong to the superfamily of HIT proteins, which are characterized by an histidine-triad sequence motif. While the eukaryotic variants hydrolyze AMP derivates and modulate transcription, the function of prokaryotic HinT proteins is less clearly defined. In *Mycoplasma hominis*, HinT is concomitantly expressed with the proteins P60 and P80, two domains of a surface exposed membrane complex, and in addition interacts with the P80 moiety.

**Results:**

An cluster of *hit*ABL genes, similar to that of *M. hominis *was found in *M. pulmonis, M. mycoides *subspecies *mycoides *SC, *M. mobile *and *Mesoplasma florum*. RT-PCR analyses provided evidence that the P80, P60 and HinT homologues of *M. pulmonis *were polycistronically organized, suggesting a genetic and physical interaction between the proteins encoded by these genes in these species. While the *hit *loci of *M. pneumoniae *and *M. genitalium *encoded, in addition to HinT, a protein with several transmembrane segments, the *hit *locus of *Ureaplasma parvum *encoded a pore-forming protein, UU270, a P60 homologue, UU271, HinT, UU272, and a membrane protein of unknown function, UU273. Although a full-length mRNA spanning the four genes was not detected, amplification of all intergenic regions from the center of UU270 to the end of UU273 by RT-PCR may be indicative of a common, but unstable mRNA.

In *Chlamydiaceae *the *hit *gene is flanked upstream by a gene predicted to encode a metal dependent hydrolase and downstream by a gene putatively encoding a protein with ARM-repeats, which are known to be involved in protein-protein interactions. In RT-PCR analyses of *C. pneumoniae*, regions comprising only two genes, Cp265/Cp266 and Cp266/Cp267 were able to be amplified. In contrast to this *in vivo *interaction analysis using the yeast two-hybrid system and *in vitro *immune co-precipitation revealed an interaction between Cp267, which contains the ARM repeats, Cp265, the predicted hydrolase, and Cp266, the HinT protein.

**Conclusion:**

In the *Mollicutes *HinT proteins were shown to be linked with membrane proteins while in the *Chlamydiaceae *they were genetically and physically associated with cytoplasmic proteins, one of which is predicted to be a metal-dependent phosphoesterase. Future work will elucidate whether these differing associations indicate that HinT proteins have evolved independently or are indeed two hotspots of a common sphere of action of bacterial HinT proteins.

## Background

The detection of an unusual, highly conserved sequence motif "His-phi-His-phi-His-phi-phi" (phi representing hydrophobic amino acids) in a variety of organisms of all kingdoms led to the definition of a new family of proteins named HIT (histidine triad) [1]. This family has three main branches: the fragile histidine triad (FHit)-related proteins found in animals and fungi, which act as di-adenosine polyphosphate hydrolases and function as tumor suppressors in humans and mice [2] (although the tumor suppressing function is not dependent on ApppA hydrolysis [3]), the GalT (galactose 1 phosphate uridylytransferase) homologues, which have a modified "His-phi-His-phi-Gln" motif, which transfers nucleoside monophosphates to phosphorylated secondary substrates rather than hydrolyzing them [4], and the Histidine-triad nucleotide binding (HinT) homologues, which in eukaryotes are intracellular receptors and hydrolases of purine mononucleotides [5]. Although HinT homologues are found in all kingdoms and this family is the most ancient and widespread branch of the HIT proteins, the cellular function, the substrates and the interaction partners of HinT proteins are largely unknown. In prokaryotes, knowledge of HinT proteins is generally restricted to sequence analyses. In the cell wall-less prokaryote *Mycoplasma hominis*, the cytoplasmic HinT protein interacts with a surface localized membrane complex by binding to the P80 moiety. Interestingly, the genes encoding P80 and P60, the domains of the membrane complex, form an operon with the HinT gene [6]. The identification of a homologous *hit *locus in *M. pulmonis *and access to several sequenced prokaryotic genomes enabled us, in this study, to hypothetically identify interaction partners and thus propose a functional role for HinT in bacteria with small genomes.

## Results

To find out more about the function of prokaryotic HinT proteins we first analyzed the *hit *loci of bacteria with a restricted genome, with the view that they might represent a model for organisms possessing the minimal genetic make-up essential for life as a free-living organism. As polycistronically organized genes often encode proteins that are functionally related (e.g. in a protein-complex formation or as part of a common pathway) we ran a search of known genome sequences for genes closely neighbouring or overlapping the *hit *gene. Species from the *Mollicutes *and the *Chlamydiaceae *fulfill these requirements and were thus analyzed.

*Mollicutes *are phenotypically distinguished from other bacteria by their minute size and total lack of a cell wall. They have evolved as a branch of gram-positive bacteria by a process of reductive evolution. The significant genome "condensation" (for example the genome of *M. genitalium *is only 580 kbp) was made possible by adopting a parasitic behavior. The primary habitats of human and animal mycoplasmas are the mucous surfaces which they colonize during infection [7]. While the intracellular localization of *Mollicutes *in insect tissues is well established, cell entry of human or animal mycoplasmas seems to be rare and possibly mediated by a site-directed, receptor-mediated event found in chlamydia [8]. However, *Chlamydiaceae *have a number of features in common with mycoplasmas: their small genomes predict a limited metabolic capability, their major target tissue are mucous membranes and they also cause ocular and sexually transmitted diseases. In contrast, the chlamydia are obligate-intracellular bacteria that undergo a unique developmental cycle in which is an alteration in size between the small metabolically inactive infectious elementar body and the relatively large, metabolically active reticular body which is adapted for intracellular growth [9]. Bearing in mind, that in *M. hominis *the HinT-interacting protein is a secreted antigen and thus predicted to play a role in the pathogenicity of mycoplasmas we expand this analysis to include a family of obligate-intracellular organisms known to secrete antigens in the inclusion body in the infected cell.

### Organization of the *hit*-locus in *Mollicutes*

**Figure 1:**
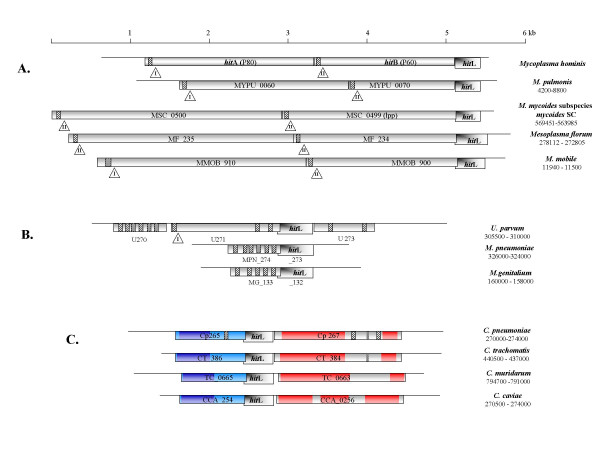
**Schematic of the *hit *loci in *Mollicutes *and *Chlamydiaceae*. **A schematic representation of genes within the *hit *loci of the following species: A) *M. pulmonis*, *M. mycoides *subsp. *mycoides *SC, *Mesoplasma florum *and *M. mobile *containing genes homologous to *hit*ABL of *M. hominis*; B) *U. parvum*, *M. pneumoniae *and *M. genitalium*, each containing a gene upstream of *hit*L predicted to encode an integral pore-forming protein; and C) *C. pneumoniae*, *C. trachomatis*, *C. muridarum *and *C. caviae *containing *hit*L flanking genes predicted to encode, upstream, a protein with the signature sequence of a metal-dependent hydrolase (light blue region), with an OB nucleic acid binding fold (dark blue region) and, downstream, a protein with ARM repeats (red regions), which are known to mediate protein-protein interactions. Triangles indicate signal peptidase recognition sites of SPase I and SPase II. Transmembrane regions are depicted as striped regions. The position of the RGD tri-peptide is marked by a dotted region.

*Hit *loci with a genomic organization comparable to that of *M. hominis *[6], with the *hit*AB genes encoding two membrane proteins and *hit*L encoding the cytoplasmic HinT, were detected in nearly half of the mollicute genomes analyzed (Figure 1A). The highest similarity was found with the *hit *locus of *M. pulmonis*. The MYPU_0080 encoded protein had 44.3 % identity to the *M. hominis *HinT protein, and the predicted MYPU_0060 and MYPU_0070 proteins were 23.8 % and 26.6 % identical to the membrane proteins P80 and P60 of *M. hominis*, which were encoded by *hit*AB. MYPU_0060 had structural features similar to those of P80, with an amino-terminal signal sequence with a predicted signal peptidase I (SPase I) cleavage site and a predominantly alpha-helical structure [10]. MYPU_0070 began with an amino-terminal signal sequence of transmembrane helix (from AA 5 to AA 20) and a signal peptidase II recognition site with a lipoprotein attachment site at position 27. Thus, MYPU_0070 of *M. pulmonis *appears to encode a P60 homologue, a cysteine-anchored lipoprotein. The order of genes within the *hit *loci of *M. mycoides *subsp. *mycoides *SC (MSC), *Mesoplasma florum *(MF) and *M. mobile *(MMOB) was similar with two genes predicted to encode membrane proteins and a downstream *hit*L gene. The similarities between the predicted sequences of MSC_0500, MF_235 and MMOB_910, and P80 of *M. hominis *were quite low and the proteins would be significantly larger than P80. While MMOB_910 contained a signal peptidase I recognition site, like P80, MSC_0500 and MF_235 were pro-lipoproteins with an amino-terminal SPase II recognition sequence. Only MSC_500 was predicted to have a predominantly alpha-helical structure, MF_235 being predicted to have a secondary structure of alternate alpha helical and beta sheet regions and MMOB_910 to consist mainly of beta sheets (data not shown). The proteins encoded by the gene next to *hit*L had little similarity with P60 of *M. hominis *when the whole sequence was compared. However, when the P60 protein region from AA 164 to AA 177 (CS1; ELQKMLLAKLYLLK) was used, identities of 43 % (MMOB_900) to 57% (MYPU_0070) were detected and scrutiny of the sequence from AA 226 to AA 234 (CS2; LYLMKYLVE) revealed 60 % (MYPU_0070) to 70 % identity (MMOB_900). The corresponding proteins of *Mesoplasma florum *and *M. mycoides *subsp. *mycoides *SC did not contain these conserved sequences. The different HinT homologues had the highest identity with the HinT protein of *M. hominis*, ranging from 44.3 % (MF_233) to 55.7 % (MMOB_890).

*Hit *loci with a different genomic organization were identified in the mollicutes *Ureaplasma parvum*,*M. pneumoniae *and *M. genitalium *(Figure 1B). While UU271 of *U. parvum*, the gene immediately upstream of *hit*L encoded a putative P60 homologue possessing the P60 consensus sequences CS1 (35.7 % identity) and CS2 (30% identity), it differed from all other P60 homologues in possessing a SPase I recognition sequence and two other transmembrane helices (AA 355 to AA 375 and AA 407 to AA 427). The UU273 gene, immediately downstream of *hit*L, encoded a protein with two transmembrane spanning helices, but with no significant similarity to other known proteins. The deduced protein sequence of UU270 did not have any sequence similarity with P80. With six transmembrane segments, it appeared more likely to form a pore than to be surface exposed. The organization of the *hit *locus of *U. parvum *appeared intermediate between the *hit *loci described above and those of *M. pneumoniae *and *M. genitalium*. The sequence identity between the ureaplasma HinT and the HinT proteins of *M. pneumoniae *(42.7 %) and *M. genitalium *(47.4 %) was higher than that between HinT of *U. parvum *and *M. hominis *(39.2 %), and the genes immediately upstream of *hit*L encoded proteins with seven (MPN_274) or five (MG_133) transmembrane domains (Figure 1B). MPN_274 is predicted to encode a permease of an ABC transporter [11] suggesting a comparable function in *M. genitalium *and probably also in *U. parvum*. Thus, these *hit *loci of *Mollicutes *analyzed appear to have a *hit*L gene flanked by genes that are predicted to encode membrane-anchored proteins.

As in *M. gallisepticum*, the *hit*L gene is not flanked by other genes on the same strand, also exceptions of a polycistronic organization of the *hit*L gene seem to exist within the *Mollicutes*.

### Organization of the *hit *loci in *Chlamydiaceae*

In the obligately intra cellular *Chlamydiaceae *the order of genes within the *hit *loci and the function of the encoded proteins appeared to be highly conserved, but distinctly different from that of the *Mollicutes *(Figure 1C). In all chlamydial species analyzed, the gene upstream of *hit*L encoded a putative cytoplasmic protein with the signature sequence of a metal-dependent protein hydrolase and a large number of metal binding residues (IPR003226/UPF0160), probably indicative of a phosphoesterase function, and an oligonucleotide/oligosaccharide-binding OB fold (IPR008994). The deduced protein sequence of the gene located downstream of *hit*L contained a 37–47 AA long tandemly repeated ARM repeat fold (IPR008938), which forms a right-handed superhelix and has been implicated in the mediation of protein-protein interactions [12]. Cp267 contained an RGD motif, which plays a role in cell adhesion [13]. However, the presence of RGD in a sequence alone is not sufficient to suggest a biological function for this motif [14]. The presence of two transmembrane helices (AA 314 to AA 334 and AA 428 to AA 448) suggested that it is more likely that Cp267 may interact with the bacterial cell membrane. In all other chlamydial species analyzed, the Cp267 homologues did not contain domains suggestive of membrane attachment.

### Are the *hit *loci genes co-expressed?

In bacteria, overlapping genes on the same coding strand may indicate the polycistronic organization of these genes, and co-expressed proteins are often related in function.

**Figure 2:**
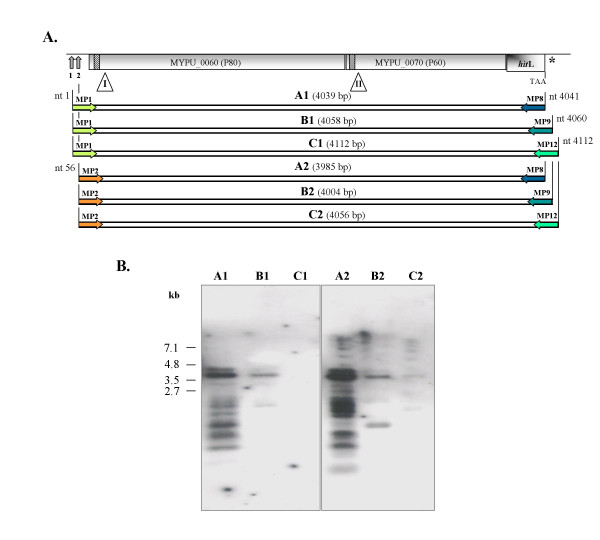
**RT-PCR analysis of *M. pulmonis*. **A. The regions amplified by RT-PCR are shown below the genomic region encompassing MYPU_0060, MYPU_0070 and MYPU_0080. The primers used and the lengths of the amplicons are indicated. B. Amplicons were separated on a 0.8 % agarose gel and subjected to Southern blot analysis, here shown for the MYPU_0070 specific probe, using digoxigenin (DIG)-labeled probes hybridizing to each of the three genes, detected using chemiluminescence. Bands of lower length as expected are degradation products. M, DIG-labeled DNA molecular weight marker VII (Roche Biochemicals).

As the *hit *locus of *M. hominis *is an operon containing three genes [6] the next step was to analyze whether the polycistronic organization of *hit *genes was conserved within the *Mollicutes *and whether this also occurred in *Chlamydiaceae*. Initially, we chose *M. pulmonis*, the species with the greatest similarity to *M. hominis*, for reverse transcription (RT) PCR analysis. To identify the likely boundaries of an mRNA containing MYPU_0060, MYPU_0070 and MYPU_0080, two different primers upstream of the MYPU_0060/*hit*A gene and three primers downstream of the *hit*L gene were used (Fig. 2A). Amplification occurred with primers binding just upstream of the predicted promoter regions 1 or 2 and just downstream of the TAA stop codon of the *hit*L gene (Fig. 2B, lanes A and B) indicating a polycistronic mRNA. Amplification was not seen with a primer hybridizing to the sequence downstream of a predicted hairpin loop structure after the TAA stop codon of *hit*L (Figure 2B, lanes C1 and C2) suggesting that the mRNA terminates at the predicted hairpin structure.

**Figure 3:**
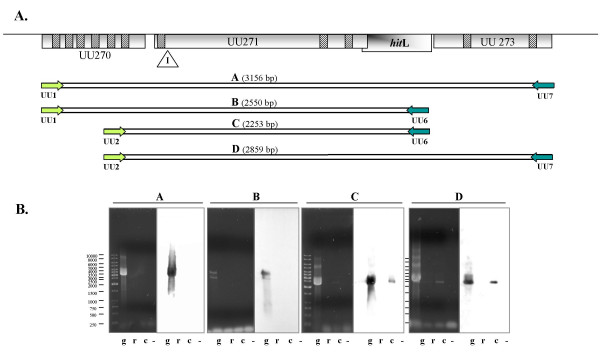
**RT-PCR analysis of *U. parvum*. **A. The positions of the different amplicons are shown below the schematic of the *hit *locus genes of *U. parvum*. The primers used (Table 2) and the lengths of the amplicons are indicated. B. The PCR products (A – D) for genomic DNA (g), cDNA (c) and RNA (r) were separated on a 0.6 % agarose gel and stained with ethidium bromide. Southern blot analysis was performed with digoxigenin (DIG)-labeled probes hybridizing to one of the four genes, (here shown for UU272 probing), and detected using chemiluminescence. M, Gene Ruler 1 kb DNA ladder (Fermentas).

Next, we examined the genes flanking *hit*L in *U. parvum*. Amplicons which spanned the intergenic regions were obtained with primers hybridizing to the center of UU270 and to the 3'-end of UU272 (Fig. 3C), and with primers hybridizing to the 5'-end of UU271 and to the 3'-end of UU273 (Fig. 3D). No amplification occurred with a primer hybridizing to the 5'-end of UU270 and the 3'-end of *hit*L (Fig. 3A and 3B). These data suggest that HinT is expressed with the flanking genes UU271 and UU273. These findings are in accordance with the predicted termination of transcription by a hairpin loop with a stem energy of -9.1 kcal mole^-1 ^located 33 nt downstream of the UU273 gene [15]. Of the organisms analyzed so far *U. parvum *was the most demanding organism of the *Mollicutes *in terms of DNA-free, full-length total RNA preparation. Thus the detection of a common RNA from the center of UU270 up to UU273 (Fig. 3D) may be due to an unstable RNA or indicatory for UU270 not taking part in the operon structure.

**Figure 4:**
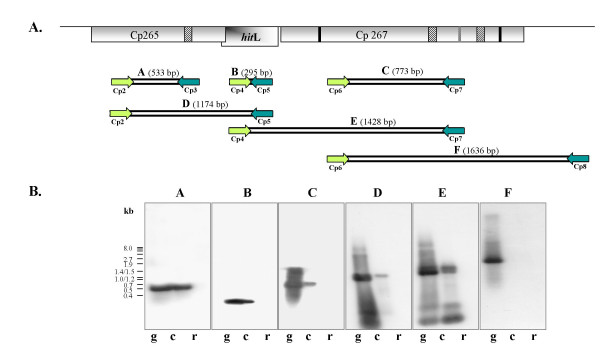
**RT-PCR analysis of *C. pneumoniae. ***A. Below the schematic of the *hit *locus of *C. pneumoniae *the positions of the PCR amplicons (A – F) are shown. Genomic DNA (g), cDNA (c) and RNA without RT reaction (r) are used as templates. B. The PCR products were separated on a 0.8 % agarose gel and subjected to Southern blot analysis with DIG-labeled probes hybridizing to Cp265 (A, D), Cp266 / *hit*L (B, E) Cp267 (C, F) with visualization using chemiluminescence. In E, the signals of lower length as 1.4 kb may be due to primer dimerization. M, DIG-labeled DNA molecular weight marker VII (Roche Biochemicals)

To characterize the organization of *hit *loci genes within the *Chlamydiaceae *we analyzed the locus in *Chlamydophila pneumoniae*. The genes Cp266 (encoding HinT), Cp267 and Cp268, which is predicted to encode a solute symporter family protein [16], were predicted to comprise an operon. No amplification occurred when a primer pair that hybridized upstream of Cp265 and downstream of Cp267 was used (data not shown). Amplification was detected using primers binding within each gene (Figure 4A,B,C) and with primers binding within Cp265 and Cp266 (Fig. 4D) and within Cp266 and Cp267 (Fig. 4E). The predicted co-expression of Cp267 and Cp268 downstream of which several putative transcription terminators were detected, was not supported by RT-PCR analysis (Fig. 4F), with amplification being obtained when using genomic DNA as a template but not with cDNA.

### Interactions between the proteins encoded by the *hit *locus of *C. pneumoniae*

**Figure 5:**
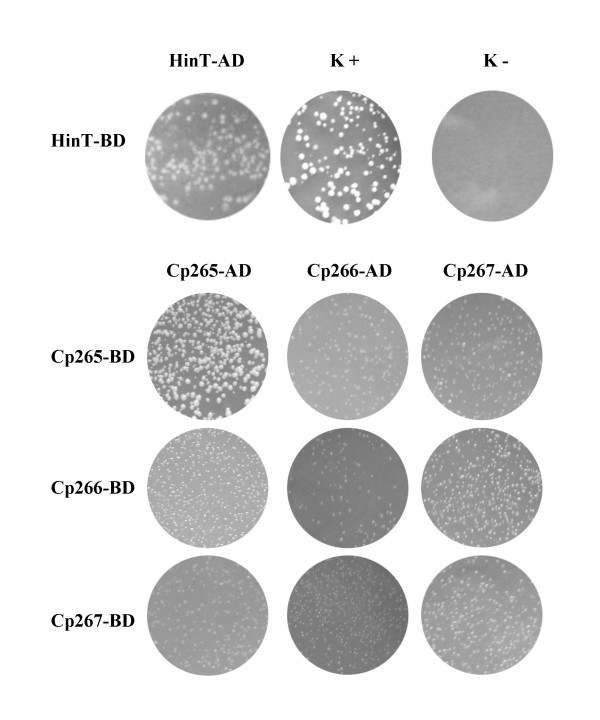
**The yeast-two hybrid assay. **AH109 were co-transformed with the pGADT7 (AD) and pGBKT7 (BD) constructs containing *hit*L of *M. hominis*, Cp265, Cp266 or Cp267 from *C. pneumoniae*, pGADT7-T, expressing the SV40 large T-antigen and pGBKT7-53, expressing the murine p53 protein (K+), or pGADT7 and pGBKT7 without fusion partners (K-). Yeast cultures were incubated at 30°C on histidine-deficient agar plates for 4 days.

**Figure 6:**
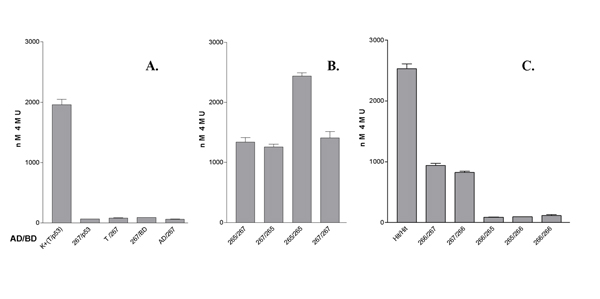
**Quantitation of β-galactosidase activity. **In yeast cells, co-transformed with pGADT7 (AD) and pGBKT7 (BD) constructs as indicated, β-galactosidase activity was quantified using 4-methylumbelliferyl-galactopyranoside (MUG) hydrolysis to the fluorescent molecule, 4-methylumbelliferone (4 MU). The fluorescence of 4 MU was excited at 360 nm and emission measured at 460 nm.

As the stability of an RNA is increased in regions with pronounced secondary structures, such as transcriptional terminators, and is often decreased in regions lacking such protective structures [17], partial degradation of the chlamydial mRNA may have occurred. The results from RT-PCR analysis of the mRNA of CP265, Cp266 and Cp267 suggest an operon producing a transient mRNA thus suggesting a physical interaction between the products of these genes. Therefore we analyzed the interactions between Cp265, Cp266 and Cp267 using the yeast two-hybrid system [18]. The system is based on the modular organization of the transcription factor GAL4, which has a DNA-binding domain (DB) and an activation domain (AD). When GAL4 binds (via DB) to its cognate binding site, the activation domain AD is brought close to the promoter and activates transcription of the reporter genes *lacZ *and *HIS3*. In the yeast two-hybrid system these two domains, which alone are not able to activate transcription, are separately cloned into pGADT7 (AD) and pGBKT7 (BD) and expressed in fusion with the proteins under investigation. Growth of a histidine dependent yeast strain on histidine deficient media will only occur after transformation with both plasmids if the AD and DB domains are apposed by an interaction between the fusion proteins, which will result in transcription of the reporter genes. The plasmids pGADT7-T and pGBKT7-53, which encode the SV40 large T-antigen and the murine p53 protein, respectively served as a positive control (Fig. 5 K+; Fig. 6A).

HinT is known to dimerize [19,20]. We introduced the coding region of *M. hominis *HinT into both pGADT7 and pGBKT7 and transformed the histidine dependent yeast strain AH109 with them. Quantification of transcriptional activation of the reporter gene was achieved using a liquid β-galactosidase assay measuring the hydrolysis of 4-methylumbelliferyl-β, D-galactopyranoside (MUG), yielding the fluorescent molecule 4-methylumbelliferone (4 MU). As expected, dimerization of the fused HinT peptides led to apposition of the AD and DB domains generating a functional transcription factor. The transcription of the reporter genes resulted in growth of the yeast on histidine-deficient agar plates (Fig. 5, top row, left plate) and production of β-galactosidase (Fig. 6C). Next we analyzed whether the proteins encoded by the *hit *locus of *C. pneumoniae *interacted. The coding regions of Cp265, Cp266 and Cp267 were introduced into pGADT7 and pGBKT7 and expressed in yeast in all possible combinations. As shown in Figure 5, large colonies, comparable in size to those of the positive control, were produced by yeast that expressed Cp265 fused to both the binding and the activation domain. This suggested a strong interaction between the molecules of the Cp265 protein. All other combinations of plasmids encoding Cp265, Cp266 and Cp267 only led to the growth of small colonies, a finding that was difficult to interpret. To differentiate between strong and weak interactions, the β-galactosidase assay was used. As shown in Fig. 6, the measurement of β-galactoside activity indicated an interaction between Cp267 and Cp265, Cp266 and Cp267 itself, independent of the fusion partner (the AD or DB domains of the GAL4 protein). Surprisingly, dimerization of Cp266 (HinT) was not detected, although Cp265 interacted strongly with itself. Cp267 did not activate transcription by itself (Fig. 6A. 267/BD and 267/AD) nor did it interact with the unrelated protein SV40 large T antigen (T/267) or murine tumor suppressor p53 (267/p53) to activate transcription, as in all these cases the β-galactosidase activity was at background levels (Fig. 6A).

### Immune co-precipitation of Cp265, Cp266 and Cp267

**Figure 7:**
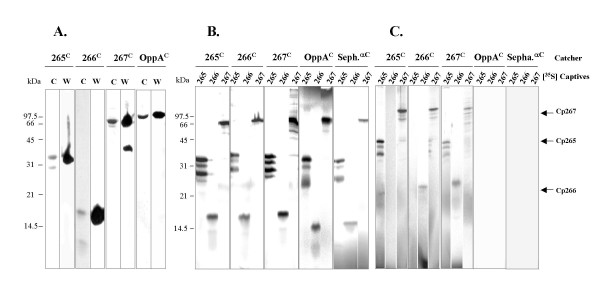
**Immune co-precipitation. **A. Affinity purified proteins, Cp265 (265^C^), Cp266 (266^C^), Cp267 (267^C^) and OppA^C^, were analyzed on 15 % SDS-polyacrylamide gels by Coomassie staining (C) or on Western blots by immunostaining with an anti-Protein C antibody (W). [^35^S]-labeled Cp265, Cp266 and Cp267 (Captives) were incubated at room temperature for 1 h with Cp265^C^, Cp266^C^, Cp267^C ^and OppA^C ^(Catcher). Immune complexes were precipitated with anti-Protein C-matrix, and the supernatants (B.) and precipitates (C.) analyzed on 15 % SDS-polyacrylamide gels by autoradiography using a phosphorimager. As a negative control, the [^35^S]-labeled proteins were subjected to the Protein C-matrix without a catcher (Sepha.^αC^). M, prestained low-molecular weight marker (Biorad)

To confirm the findings derived from the two-hybrid analyses we performed protein-binding assays *in vitro*. Protein C-tagged Cp265, Cp266 and Cp267 were expressed in *E. coli *and purified by affinity chromatography (Fig. 7A). Each protein was then incubated with equal amounts of [^35^S]-labeled Cp265, Cp266 or Cp267 *in vitro *and precipitated with anti-Protein C antibodies. As shown in Fig. 7B, the supernatants of the different samples contained comparable amounts of the respective isotope labeled proteins. As expected the [^35^S] labeled Cp proteins alone were neither precipitated by unrelated membrane proteins, such as Protein C-tagged OppA of *M. hominis *nor by the anti-Protein C-sepharose itself (Fig. 7C). In contrast, an interaction of Cp266 with Cp267 was suggested by co-precipitation of significant amounts of Cp266 [35*S*] with Cp267^C^, and Cp267 [35*S*] with Cp266^C ^(Fig. 7C). The other findings from the two-hybrid analyses were also confirmed as both Cp265 and Cp267 were found to dimerize and there was no interaction between Cp265 and Cp266. Interestingly dimerization of Cp266 (HinT) which was not detected by the two-hybrid system, was demonstrated in this co-precipitation assay.

Thus, we can conclude that the chlamydial proteins Cp265, Cp266 (HinT) and Cp267 have a tendency to dimerize and Cp267 interacts with both Cp265 and Cp266.

## Discussion

A remarkable feature of HinT proteins is their ubiquity. They are found in all kingdoms ranging from *Mycoplasma genitalium*, the smallest prokaryote, to one of the most complex eukaryotes, the human [1,19]. However the function of this homo-dimer in the cytoplasm of these different organisms has only been closely examined in eukaryotes, where it has been characterized as an intracellular receptor for purine ribonucleotides [4,5], especially for hydrolases of 5'-monophosphoramide substrates such as AMP-lysine [21], with the histidine triad motif forming the α-phosphate binding site [20]. HinT has been shown to associate with the protein microphthalmia, an important transcription factor that controls growth and function in mast cells and melanocytes [22], to interact with the human cyclin-dependent kinase 7 (Cdk7), a subunit of the RNA polymerase II C-terminal domain kinase Cdk7/Kin28 [23], and to act as a positive regulator of the yeast Cdk7 homologue Kin28 [21]. That it may not be the key regulator of Cdk7 activity was recently suggested by analysis of HinT^-/- ^knock-out mice [24]. This led to the hypothesis that eukaryotic HinT homologues are involved in the regulation of transcriptional processes by hydrolyzing related adenylyl-modified proteins [25].

The observation that HinT homologues are ubiquitous and that they are even encoded by the smallest bacterial genomes, suggests that HinT was present at the cellular root of the tree of life and that its preservation is advantageous for the survival of cells [20]. However the function of bacterial HinT homologues seems to be quite different from those of eukaryotes. In *M. hominis *HinT had been demonstrated to interact both physically and genetically with a surface-localized membrane complex by binding to the P80 domain [6]. The data presented here suggest that the co-expression of HinT with membrane proteins is prevalent within the *Mollicutes*. Comparable organization of the *hit*-loci was found in *Mycoplasma hominis, M. pulmonis, M. mycoides *subsp. *mycoides *SC, *M. mobile *and *Mesoplasma florum*. Analysis of further genomes will most likely lengthen this list. A genomic DNA fragment of *M. bovis *encoding the amino-terminal end of a P80 homologue has been sequenced and this homologue is predicted to possess an SPase I cleavage site. In *M. hyorhinis*, a nearly complete *hit *locus, encoding P80, P60 and HinT homologues has been found adjacent to a genomic region encoding the high affinity transport system (personal communication of Michael Calcutt, Columbia, MO, USA) [26]. The organization of the *hit*ABL genes in an operon, as recently demonstrated for *M. hominis *[6] and shown here for *M. pulmonis*, suggests polycistronic expression of the *hit *loci genes. However, it remains to be elucidated whether the encoded proteins have comparable functions to those already shown in *M. hominis*. P80 of *M. hominis *was recently shown to reside in the membrane as a precursor protein and to be secreted into the extra cellular milieu as a 10 kDa smaller antigen. Processing of P80 was suggested to be initiated by SPase I cleavage [27]. While the P80 homologues in *M. mobile *and *M. bovis *also contained signal sequences for SPase I cleavage, those in *M. mycoides *subsp. *mycoides *SC and *Mesoplasma florum *were putative pro-lipoproteins. Secretion of lipoproteins has been observed in *M. hominis *[6] and other bacteria [28], indicating that similar function may be still possible.

*M. pneumoniae *and *M. genitalium *each had only one gene adjacent to *hit*L encoding a pore-forming protein with homologies to ABC permeases, suggesting they are part of a distinct phylogenetic branch. Interestingly, in *M. penetrans *the gene upstream of *hit*L also encodes a protein of an ABC transporter, an ATPase [29]. The *hit *locus of *U. parvum*, which contained a P60 homologue and a gene encoding a permease, may have functions that are a hybrid of those of *M. hominis *and the *M. pneumoniae *groups. RT-PCR analyses infer the presence of a mRNA encoding HinT and UU271, a P60-homologue, and a membrane protein of unknown function. Thus, the interaction of HinT with membrane proteins seems to be a common phenomenon in *Mollicutes*.

A quite different situation was detected in the *Chlamydiaceae*. Although we were not able to establish definitely the presence of a polycistronic mRNA derived from the three genes comprising *hit *locus, the overlap of the *hit*L gene with the upstream gene, as well as the conserved order of the three genes within the *hit *loci of *Chlamydiaceae*, suggested a relationship between the gene products. Analysis of Cp265, Cp266 (HinT) and Cp267 in the yeast two-hybrid system and immune co-precipitation assays confirmed our hypothesis that these proteins interacted and demonstrated that they form homo-dimers. Cp267, a protein with large areas of ARM repeats, which are known to mediate protein-protein interactions [30], was shown to interact with HinT, a protein known in eukaryotes to influence transcriptional activation, as well as with Cp265, a putative metal-dependent hydrolase with a binding fold for short, single stranded nucleic acids. These findings suggest that in *Chlamydiaceae*, the function of HinT may be more closely related to intracellular processes than to interactions with the extracellular milieu, as suggested by the findings on *Mollicutes*.

## Conclusion

The data presented here demonstrate that HinT proteins of the *Chlamydiaceae *associate with probable cytosolic proteins likely to function in the regulation of cellular processes, such as nucleotide metabolism. This function would be similar to that of homologues in eukaryotes, where HinT has been shown to influence transcription. The finding that HinT proteins of the *Mollicutes *interact both physically and genetically with membrane proteins may reflect a phylogenic shift towards a different function in this group, or may indicate an additional function of bacterial HinT proteins. Our future work will focus on decoding the intra- and extra-cellular processes that bacterial HinT proteins are involved in.

## Methods

### DNA manipulations

All routine DNA manipulation techniques, including plasmid preparation, restriction endonuclease analysis, ligation and transformation of *E. coli *were performed as described by Sambrook *et al*. [31] or according to the manufacturers' recommendations (Qiagen, Hilden, Germany, and Roche Applied Science, Mannheim, Germany).

### Bacterial strains and plasmids

The plasmids pXB (Roche Applied Science, Mannheim, Germany), pGADT7 and pGBKT7 (BD Bioscience Clontech, Palo Alto, USA) were used as expression vectors for the heterologous expression of Cp265, Cp266 and Cp267. The pXB plasmids were propagated in *Escherichia coli *DH5α F'(Life Technologies), pGADT7 and pGBKT7 in yeast AH109 cells (BD Biosciences Clontech, Palo Alto, CA USA).

### Sequence analysis

Analysis of the DNA and protein sequences and the design of oligonucleotides were facilitated by the Lasergene software (DNA Star Inc., Madison Wisc.).

The genomes listed in table 1 were analyzed using the web site of NCBI .

Computer-based prediction of transcription termination was performed using the GeneBee service (online ). Distant relation-ships between HinT protein sequences of the analyzed *Mollicutes *were determined by using the BLAST method through Molligen 1.4 [32]. Conserved domains of proteins were predicted using the Conserved Domain Database (CDD) or the InterPro service (, ).

### RT-PCR

RNA was prepared from exponential phase cultures of *M. pulmonis *and *U. parvum *as described by Chomczynski and Sacchi [33]. *Chlamydophila pneumoniae *respiratory isolate GiD was used for all experiments [34]. Chlamydial RNA was prepared from HEp-2 cells 2 days post-infection as described by Mölleken et al. (Mölleken et al., manuscript in preparartion). Before use as a template for RT-PCR, contaminating traces of DNA were digested with DNase I as described previously [35]. Overlapping regions of the *Ureaplasma *and *Chlamydophila *genes were amplified using the Expand Template PCR system (Roche Applied Science, Mannheim, Germany) by standard PCR conditions (initial cycle of 5 min at 95°C; 35 cycles of 1 min at 95°C, 1 min at 50°C (*U. parvum*) or 58°C (*C. pneumoniae*), 2.5 min at 68°C). The *hit *operon of *M. pulmonis *was amplified using the Expand Long Template PCR system by standard PCR conditions (initial cycle of 5 min at 95°C; 10 cycles of 1 min at 95°C, 1 min at 44°C, 4 min at 68°C; addition of 0.5 U polymerase; then 20 cycles of 1 min at 95°C, 30 sec at 44°C, 4 min (+ 20 sec per cycle) at 68 °C). Southern blot analysis was performed as described previously [6].

### Construction of plasmids

The regions encoding Cp265, Cp266 and Cp267 in *C. pneumoniae *GiD [34] were amplified by PCR with the primers Cp1 to Cp6 (Table 2). The amplicons were cut at the primer-introduced restriction endonuclease cleavage sites for *Bam*HI and *Sac*I, and ligated in-frame into the expression vector pGADT7. The *Eco*RI/*Sac*I inserts in the pGADT7 constructs were then ligated in-frame into *Eco*RI/*Pst*I-digested pGBKT7.

For heterologous expression of the protein-C tagged proteins, CP265^C^, Cp266^C ^and Cp267^C^, in *E. coli*, the Cp inserts of the respective pGADT7 plasmids were digested with *Sac*I, the ends bluntened by incubation with 3 units S1 nuclease (Amersham Bioscience, Freiburg, Germany) for 30 min at 37°C and subsequently cut with *Eco*RI. The blunt-end/*Eco*RI digested Cp265, Cp266 and Cp267 fragments were cloned in-frame into the blunt-end/*Eco*RI digested plasmid pXB and propagated in *E. coli *Dh5α F'.

All plasmid constructs were confirmed by DNA sequencing (ABI 373 A machine) [36].

### Expression and purification of Cp265^C^, Cp266^C ^and Cp267^C^

One liter of LB broth (Gibco BRL, Life Technologies Inc., Gaithersburg, USA) containing ampicillin (100 μg/ml) was inoculated with the respective *E. coli *DH5α F' clone and incubated for 16 h at 37°C with vigorous shaking. The cells were harvested by centrifugation (15,000 × g, 20 min, 4°C) and frozen at -70°C. After thawing on ice, the cells were resuspended in 40 ml buffer A (120 mM NaCl, 1 mM CaCl_2_, 20 mM Tris-HCl pH 7.5) and disrupted by three repeated freeze-thaw cycles in liquid nitrogen, followed by three bursts of sonication on ice (5 min bursts at 95 W with a 1 min cooling period between each burst). Insoluble material was sedimented (15,000 × g, 20 min, 4°C) and the supernatant was transferred to an anti-Protein C affinity matrix (Roche Applied Science, Mannheim, Germany). Bound proteins were eluted from the anti-Protein C affinity matrix with 2 mM EDTA.

### SDS-PAGE and immunostaining of proteins

Proteins were separated on 12 or 15 % polyacrylamide gels [37], transferred to nitrocellulose (Schleicher and Schüll, Dassel, Germany) using a semi-dry blotting apparatus (Phase, Mölln, Germany) [38], and immunostained using anti-Protein C peroxidase (Roche Applied Science, Mannheim, Germany), anti HA-antibody (pGADT7) or anti c-myc antibody (pGBKT7) (BD Bioscience Clontech, Palo Alto, USA).

### Immune co-precipitation assay

The [^35^S]-labeled Cp265, Cp266 and Cp267 proteins were generated from pGADT7 and pGBKT7 fusion vectors by *in vitro *translation using a T7 coupled transcription/translation system in the presence of [^35^S]-methionine (Promega, Madison, USA). Two micrograms of purified Protein C-tagged protein were incubated with 10 μl of the [^35^S]-labeled protein in buffer B (20 mM Tris/HCl (pH 7.5), 75 mM KCl, 0.1 mM EDTA, 2.5 mM MgCl_2_, 0.05 (v/v) % Igepal, 2 mM DTT, 10 (v/v) % glycerol and 2 mM CaCl_2_) for 1 h [39]. Thereafter, a 5 μl aliquot of a slurry of the anti-Protein C matrix, equilibrated in buffer B containing 5 μg BSA, was added to each sample and the mixture further incubated for 1 h. The Protein C-matrix was washed eight times in buffer B, resuspended in 20 μl of SDS-PAGE sample buffer and heated at 95°C for 5 min. The proteins were then analyzed by 15 % SDS-PAGE and autoradiography using a phosphorimager (Fujifilm FLA 3000).

### Yeast two-hybrid assays

The MATCHMAKER Gal4 two-hybrid system (BD Bioscience Clontech, Palo Alto, USA) was used for interaction assays. AH109 cells were co-transformed with the Cp265, Cp266 or Cp267 expressing pGADT-T7 and pGBKT7 plasmids, grown in small scale cultures and plated onto histidine deficient agar plates, followed by a β-galactosidase colony lift filter assay. All procedures were carried out as outlined in the BD Bioscience Clontech protocol. The β-galactosidase assay was performed after the inoculation of 7.5 ml YPD Medium with 0.5 ml overnight culture in selective medium and incubation for five hours at 30°C until the culture reached an OD_600 _of 0.4 to 0.6. The cells were harvested by centrifugation at 14,000 × g for 1 min and re-suspended in 300 μl Buffer Z (Na_2_HPO_4_+ 7 × H_2_O, NaH_2_PO_4 _+ 4 × H_2_O, KCl, MgSO_4 _× 7 H_2_O, pH7.0). Lysis of the cells was achieved with four cycles of freezing in liquid nitrogen and thawing at 37°C. Soluble and insoluble components were separated by centrifugation at 14000 × g for 2 min. The β-galactosidase activity was measured using a FluorAce™ β-galactosidase Reporter Assay kit (BioRad, Hercules, USA).

## Authors' contributions

MH carried out the immunoassays and Yeast TwoHybrid assays. BH carried out the molecular genetic studies. BH and JH participated in the design of the study and drafted the manuscript. All authors have read and approved of the final manuscript.

## Figures and Tables

**Table 1:** Organisms used for genome sequence analyses

*Chlamydophila caviae *GPIC	NC_003361
*Chlamydia muridarum*	NC_002620
*Chlamydophila pneumoniae *AR39	NC_002179
*Chlamydia trachomatis *D/UW-3/CX	NC_000117
*Mesoplasma florum *L1	NC_006055
*Mycoplasma gallisepticum *R	NC_004829
*Mycoplasma genitalium *G-37	NC_000908
*Mycoplasma mobile *163 K	NC_006908
*Mycoplasma mycoides spp.mycoides SC *str. PG1	NC_005364
*Mycoplasma pneumoniae *M129	NC_000912
*Mycoplasma penetrans *HF2	NC_004432
*Mycoplasma pulmonis *UAB CTIP	NC_002771
*Parachlamydia sp. *UWE25	NC_005861
*Ureaplasma parvum *serovar 3 str. ATCC 700970	NC_002162

**Table 2:** Primers used in PCR

**Primer**	**Length**	**Sequence (5'-3')**
MP1	27	ATTTATTTTTGATTACTATTTTTGAAC
MP2	23	AATTTGTTTTATTATTTTGTTTA
MP8	20	TTAATGTTCATGTCCTTCTT
MP9	21	AATCCAGAAAGTTTTTTTATT
MP12	24	ATTTGTTATTGCTAAAAGATTGTA
		
UU1	22	ATGATCAATAAAAACAAAAAAT
UU2	19	GCGCCGTTAGTGATGTTAG
UU6	32	TGAGCTCTTCCATATTTTTTGATTATACCATA
UU7	23	AAAAACGAATTGCCTCTATGTAT
		
Cp1	21	CATGATGCGCGTGGAGTAGGT
Cp2	25	TCCAAGAAGCATTGGTACTCACGAT
Cp3	22	CGGTTTCCATGGCTTCTCTGAC
Cp4	29	TTATCGATGGATTGATAGATTGTGAAAAG
Cp5	22	GGACGCCCACCTAAAAGATGAA
Cp6	21	TTTCGCTTGGTCCCTCTCCTG
Cp7	25	GCTCTTTCAATATCTTCACGGCTCA
Cp8	28	TACGGTGCTGAGCTTAAAGATAGAAATG
		
Cp_265 up	27	ATGGATCCTAATGGAGGATTGGCTAAG
Cp_265 low	29	TTGAGCTCTCATATGATCCCTCTATCTTG
Cp_266 up	27	TTAGGATCCTCATGCCTACGTGCTTTG
Cp_266 low	29	TTTGAGCTCATCAGGCTATAGCACCTAAA
Cp_267 up	23	ATTGGATCCCCATGTTCGGCTCG
Cp_267 low	30	TTAGAGCTCACTTGAAAATAGAGAAAAGAG
		
Cp1 BamHI	27	ATGGATCCATGGAGGATTGGCTAAG
Cp2 BamHI	27	TTAGGATCCATGCCTACGTGCTTTG
Cp3 BamHI	23	ATTGGATCCATGTTCGGCTCG
